# Preparation and Catalytic Performance Study of TiO_2_-Based Composite Photocatalysts Containing Natural Green CQDs

**DOI:** 10.3390/molecules31111898

**Published:** 2026-06-01

**Authors:** Faxue Ma, Zhen Ma, Xiangju Wu, Xueqing Zhu, Yuguang Lv, Yukang Sun

**Affiliations:** 1 College of Engineering, Huanghe S&T University, Zhengzhou 450046, China; zzbb12388@163.com (Z.M.); 16692882324@163.com (X.W.); yuguanglv@163.com (Y.L.); 2 National Engineering Research Center of Wheat and Corn Further Processing, School of Food Science and Technology, Henan University of Technology, Zhengzhou 450001, China; 3 State Key Laboratory of Green Building Materials, China State Building Materials, Research Institute, Chaoyang District, Beijing 100024, China

**Keywords:** natural green, TiO_2_-based, composite, photocatalyst, catalytic performance

## Abstract

Semiconductor photocatalysis technology is a simple, efficient, and low-cost method for environmental pollution remediation. As a promising photocatalyst for oxidative degradation, titanium dioxide (TiO_2_) demonstrates the capability to address energy shortages and environmental pollution issues. In this study, orange peel was used as the raw material to synthesize a (TiO_2_-CdS-C_3_N_4_-CDs) TCCC composite photocatalyst containing natural green carbon dots via a one-pot hydrothermal method for the first time. This catalyst was applied to the catalytic degradation of multiple dye molecules (Rhodamine B, Methylene Green, Reactive Brilliant Blue KN-R) and quinolone antibiotic (Ciprofloxacin, CIP) as well as tetracycline antibiotic (Tetracycline, THC). Meanwhile, it provides more adsorption sites for target pollutants and loads electron reservoirs (CDs) on the TCC surface, promoting the separation of photogenerated carriers in pure TiO_2_, thereby enhancing the visible light utilization and photocatalytic activity of the material. This work expands the application scope of semiconductor photocatalysis technology and TiO_2_-based photocatalytic active substrates.

## 1. Introduction

Semiconductor photocatalysis technology, as a simple, efficient, and cost-effective strategy for pollutant degradation, has been widely employed in treating organic dyes in industrial wastewater. The principle involves irradiating semiconductor nanomaterials with light energy equal to or greater than their bandgap, enabling structural defects and dangling bonds within the materials to capture charge carriers that participate in redox reactions. It is well-established that superior photocatalytic performance hinges on high-quality substrate materials; thus, developing advanced photocatalytic materials remains a primary challenge for researchers.

In recent years, significant progress has been made in photocatalyst development. Titanium dioxide (TiO_2_), an n-type semiconductor, is regarded as one of the most promising photocatalysts for degrading organic pollutants due to its excellent photocatalytic activity, high chemical stability, and unique electronic structure [1,2,3,4,5]. However, its practical applications are limited by drawbacks common to traditional semiconductors, including a wide bandgap, weak visible-light absorption, and low charge-carrier migration efficiency. Therefore, exploring effective strategies to enhance the activity of TiO_2_-based photocatalysts is crucial. Constructing heterojunctions represents one of the current research frontiers.

Cadmium sulfide (CdS), an excellent n-type transition-metal sulfide, features sulfur vacancy defects on its surface that provide abundant adsorption sites for water molecules, accelerating their dissociation [6,7,8,9]. Coupling CdS with TiO_2_ not only improves hydrophilicity but also promotes charge-carrier transfer on the material surface, enhancing photocatalytic activity [10]. Although heterostructures facilitate electron–hole separation, recombination rates may still exceed expectations during practical implementation, limiting photocatalytic efficiency and broad applicability.

Graphitic carbon nitride (g-C_3_N_4_), a metal-free photocatalytic material, has emerged as a focal point in energy and environmental research due to its large specific surface area, straightforward synthesis, and tunable bandgap [11]. In this study, a hybrid heterojunction TCC photocatalytic material with a high specific surface area and abundant oxygen vacancies (OVs) was ingeniously constructed via an in situ-induced synchronous synthesis strategy using urea. Under high-temperature conditions, the organic semiconductor g-C_3_N_4_, generated by urea polycondensation, was in situ grown to form an organic/inorganic heterojunction with TiO_2_ and CdS. The strong coupling effect at the interface effectively separates electron–hole pairs, optimizing the utilization of photoinduced electrons. Concurrently, ammonia generated during urea decomposition enriches OVs on the TiO_2_ surface through reduction reactions [12]. Additionally, the large specific surface area of g-C_3_N_4_ provides additional adsorption sites for target pollutants. Carbon quantum dots (CQDs) have attracted extensive attention for their superior photobleaching resistance and excellent optical properties [13]. Serving as an electron reservoir [14], CQDs further enhance the separation of photoinduced electron–hole pairs in TiO_2_ under ultraviolet light.

In recent years, TiO_2_, CdS, and g-C_3_N_4_ have been widely investigated as typical semiconductor photocatalysts, and various binary and ternary composites such as TiO_2_–CdS, TiO_2_–g-C_3_N_4_, CdS–g-C_3_N_4_, and their ternary hybrids have been developed to address the limitations of single-component materials, including narrow visible-light response, rapid electron–hole recombination, and low specific surface area. Despite these advances, most reported multi-component photocatalysts still suffer from insufficient charge separation efficiency, limited visible-light utilization, and lack of an effective electron-trapping center, which restrict their practical applications in pollutant degradation. To overcome these bottlenecks, we rationally design and construct a novel quaternary TiO_2_–CdS–g-C_3_N_4_–CQDs (TCCC) composite photocatalyst in this work. The selection of these four components is motivated by a well-defined double Z-scheme heterojunction strategy: CdS with a narrow bandgap is coupled with TiO_2_ to extend visible-light absorption; g-C_3_N_4_ with a large specific surface area serves as a support and builds a Z-scheme charge transfer pathway to promote electron–hole separation; natural green CQDs derived from orange peel are introduced as electron reservoirs to further capture photogenerated electrons and suppress carrier recombination. Such a complex but synergistic structure integrates the advantages of each component, aiming to achieve extended light absorption range, abundant oxygen vacancies and active sites, accelerated charge migration, and significantly improved photocatalytic activity for the degradation of organic dyes and antibiotics.

Malachite green (MG) is a typical highly toxic and refractory triphenylmethane dye commonly found in aquaculture and industrial wastewater. In recent years, a variety of photocatalysts including TiO_2_-based composites, CdS-based heterojunctions, g-C_3_N_4_-based materials, and carbon quantum dot modified catalysts have been widely studied for the degradation of MG. However, traditional single and binary composite photocatalysts still face bottlenecks such as narrow light response range, high photogenerated carrier recombination rate, and low specific surface area, which lead to unsatisfactory degradation efficiency toward MG.

Therefore, in this research, a one-pot solvothermal method was employed to synthesize a TCCC composite photocatalyst. The photocatalytic activity of the material was evaluated by degrading organic pollutants under visible light. Structural characteristics and optoelectronic properties of the composite catalyst were elucidated using various characterization techniques. Finally, the reusability and practical applicability of the composite catalyst were assessed, and the reaction mechanism occurring on its surface was discussed.

## 2. Results and Discussion

### 2.1. Sample Characterization

**Figure 1:**
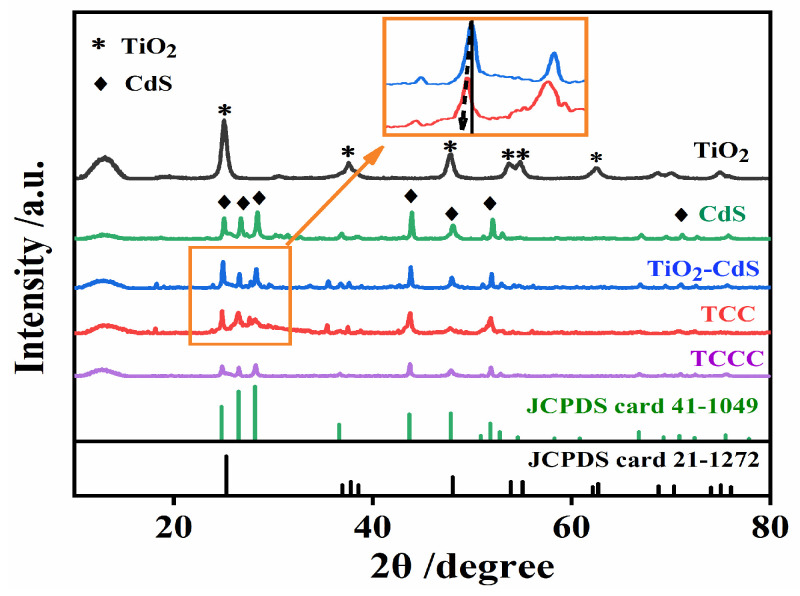
XRD patterns of different samples.

Figure 1 shows the X-ray diffraction (XRD) patterns of pure TiO_2_, CdS, TiO_2_-CdS, TCC, and TCCC. The diffraction peaks of pure TiO2 and CdS match well with anatase TiO_2_ (JCPDS No.21-1272) and hexagonal CdS (JCPDS No.41-1049), respectively. The main characteristic peaks of the composite structure at 25.28° (101), 36.94° (103), 37.80° (004), 48.04° (200), 53.89° (105), 55.06° (211), and 62.68° (204) correspond to anatase TiO_2_ (JCPDS No. 21-1272) [15]. Peaks at 26.77° (100), 28.44° (101), 43.68° (110), 47.83° (103), and 72.38° (114) correspond to hexagonal CdS (JCPDS No. 41-1049), indicating high crystallinity of the samples.

Characteristic peaks of anatase TiO_2_ and hexagonal CdS are clearly observed in TiO_2_-CdS, TCC, and TCCC composites. However, no characteristic peaks of g-C_3_N_4_ are detected in TCC and TCCC, likely due to the g-C_3_N_4_ content being below the XRD detection limit. Notably, compared with pure TiO_2_, the (100) and (002) peaks of hexagonal CdS in TCC shift to lower 2θ angles [16], which may result from interfacial interactions induced by the introduction of g-C_3_N_4_. This phenomenon suggests that the in situ-induced TCC composite structure prepared with urea as an adjuvant and raw material exhibits stronger interfacial interactions. Additionally, the characteristic peak of CDs at 21.5° in TCCC is too weak to observe [17], as the low content of CDs (molar ratio of CDs to TiO_2_ ≈ 5%) in the composite masks the peak.

A weak broad peak at approximately 12° is observed in all samples. Since this 2θ position is outside the typical range for amorphous TiO_2_ (20–35°), and a similar signal also appears in the pure CdS sample, this signal cannot be assigned to amorphous TiO_2_. This weak peak may originate from residual organic groups, unreacted precursors, or instrumental background interference rather than amorphous titanium oxide.

**Figure 2:**
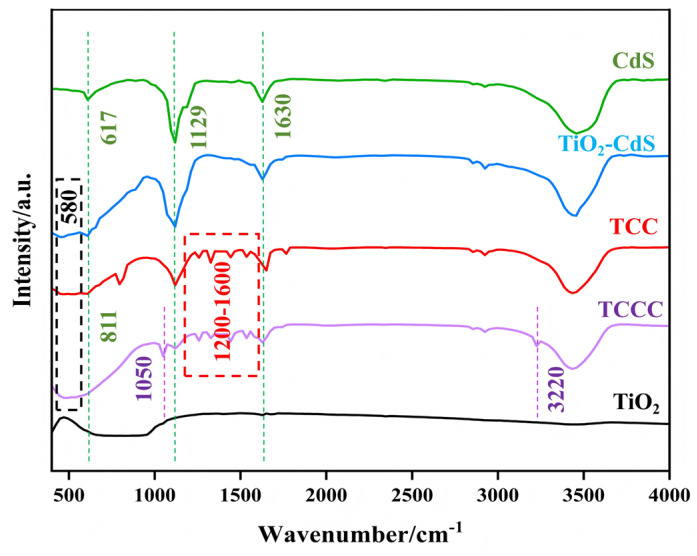
FT-IR spectra of different samples.

The framework structure and surface functional groups of samples were further analyzed by Fourier transform infrared spectroscopy (FT-IR) (Figure 2). For pure TiO_2_, the broad band at 500–1000 cm^−1^ corresponds to Ti–O stretching vibrations, while the peak at 1630 cm^−1^ is assigned to the bending vibration of adsorbed hydroxyl groups. The broad band around 3377 cm^−1^ is attributed to the stretching vibration of O–H bonds from surface hydroxyl groups and adsorbed water molecules. In TiO_2_–CdS, TCC, and TCCC composites, the band at 580 cm^−1^ is assigned to Ti–O stretching [18,19].

The peaks at 617 cm^−1^ and 1129 cm^−1^ are observed in all CdS-containing samples. The peak at 617 cm^−1^ corresponds to Cd–S stretching, while the band at 1129 cm^−1^ is attributed to S–O stretching vibrations originating from slight surface oxidation of CdS during calcination. In TCC and TCCC, the weak band at 811 cm^−1^ is characteristic of the heptazine ring mode of g-C_3_N_4_. No obvious shift in the O–H band near 3300 cm^−1^ was observed in the spectra. In TCCC, vibration peaks at 1396 cm^−1^ and 1744 cm^−1^ correspond to C=C and C-O-C bonds, respectively [20].

**Figure 3:**
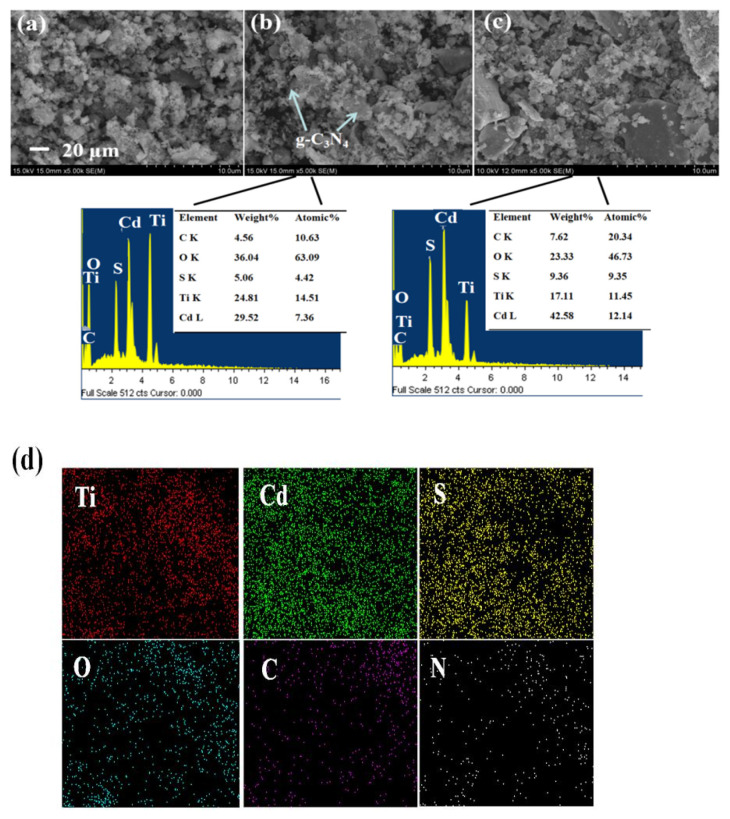
SEM images of (**a**) TiO_2_-CdS NPs, (**b**) TCC composite, and (**c**) TCCC composite; (**d**) overall EDS spectrum of TCCC.

Figure 3a–c exhibit the microstructures of CdS-TiO_2_, TCC, and TCCC. Notably, numerous aggregated particles cover the entire surface and embed within the cross-section of CdS-TiO_2_ (Figure 3a), indicating the successful formation of a TiO_2_-CdS composite. Figure 3b further confirms that TiO_2_-CdS aggregates are well dispersed and anchored on the surface of g-C_3_N_4_. Energy-dispersive spectroscopy (EDS) reveals that TCC consists of primary elements C, O, Ti, Cd, and S, further confirming the successful deposition of CdS-TiO_2_NPs [21]. The nitrogen element originates from g-C_3_N_4_ in TCC. Figure 3c demonstrates the successful incorporation of CQDs into TCC. The increased carbon content in the EDS spectrum, attributed to CQDs, further validates the successful preparation of the TCCC sample [22].

The elemental composition determined by EDS was consistent with the nominal molar ratio of TiO_2_:CdS:g-C_3_N_4_ = 1:1:2, indicating that the components were successfully introduced into the composite with good stoichiometric control.

**Figure 4:**
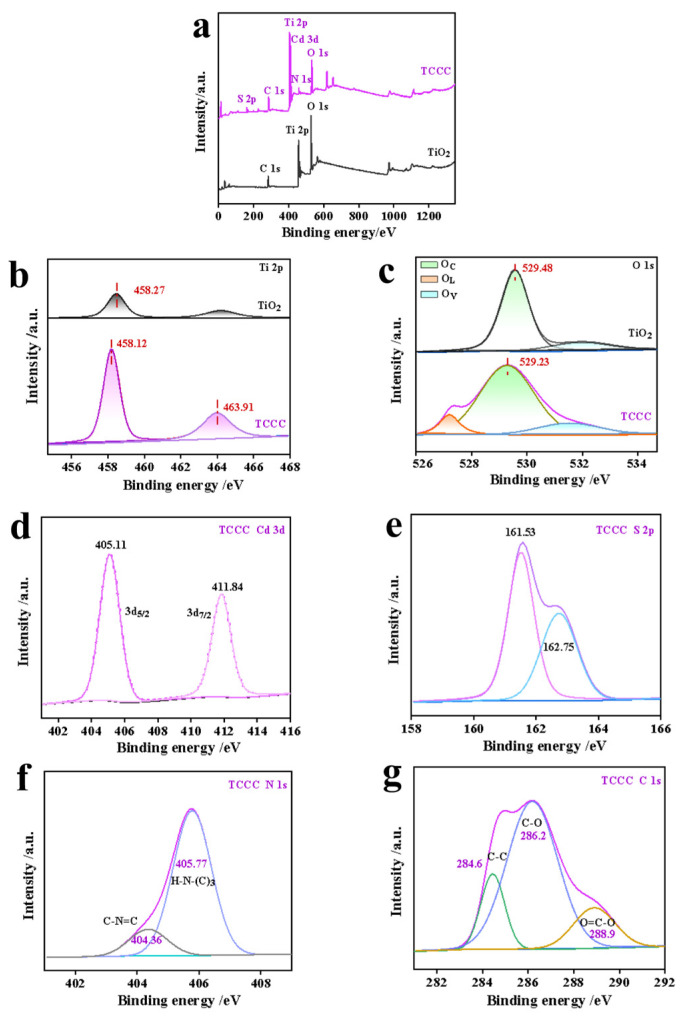
(**a**) XPS survey spectra of TCCC and TiO_2_; (**b**–**g**) high-resolution XPS spectra of Ti 2p, O 1s, Cd 1s, S 2p, N 1s, and C 1s for TCCC.

X-ray photoelectron spectroscopy (XPS) measurements were performed on different samples. Figure 4a shows the XPS survey spectra of pure TiO_2_ and TCCC, indicating that the TCCC surface contains only Ti, O, C, Cd, S, and N elements. The high-resolution Ti 2p spectrum of TCCC (Figure 4b) displays main peaks at 463.78 eV and 458.12 eV, assigned to Ti 2p1/2 and Ti 2p3/2, respectively. Compared with pure TiO_2_, the binding energies of Ti 2p in the composite sample decrease by 0.20 eV and 0.15 eV, respectively. Combined with Figure 4d,f, this shift clearly indicates that the formation of the composite alters the binding energy of Ti 2p in TiO_2_. The O 1s spectrum can be deconvoluted into lattice oxygen (OL) and oxygen vacancy (OV) characteristic peaks. The binding energy of O 1s in Figure 4c shows a similar shifting trend to Ti 2p, which can be attributed to the presence of OV [23]. The N 1s spectrum in Figure 4f is decomposed into two peaks: the peaks at 404.36 eV and 405.77 eV correspond to sp^2^-bonded N in C=N-C and H-N-(C)_3_, respectively [24]. The C 1s spectrum in Figure 4g is deconvoluted into three peaks [25]: peaks at 284.6 eV, 286.2 eV, and 288.9 eV correspond to C-C, C-O, and O=C-O, respectively. The reference peak at 284.8 eV overlaps with the C-C peak and is not separately displayed in the figure. The C 1s peak at 284.8 eV serves as a reference for amorphous carbon. The XPS results are consistent with the EDS findings.

**Figure 5:**
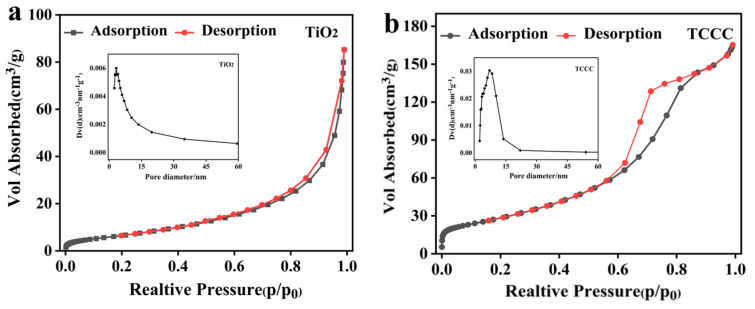
(**a**) N_2_ adsorption–desorption isotherms of TiO_2_; (**b**) N_2_ adsorption–desorption isotherms of TCCC.

The specific surface area and reactive active sites of catalysts are crucial for pollutant degradation. In this experiment, N_2_ adsorption–desorption isotherms (BET) were used to analyze the specific surface area and pore size distribution of photocatalysts. As shown in Figure 5, the adsorption isotherms of all samples belong to typical Type IV curves with H3-type hysteresis loops, indicating the presence of mesoporous structures in these catalysts. Notably, the modified TCCC sample exhibits a significantly increased specific surface area compared to pure TiO_2_. The specific surface area of TCCC is 91.5 m^2^/g, with a pore volume of 0.256 cm^3^/g, which is 4.56 times that of pure TiO2. Notably, pure g-C_3_N_4_ possesses a large specific surface area, which is beneficial for exposing more active sites and adsorbing more pollutant molecules. Although the BET data of pure g-C_3_N_4_ was not measured separately in this work, its large surface area has been widely confirmed in the previous literature. The high specific surface area of TCCC (91.543 m^2^/g) further confirms that the introduction of g-C_3_N_4_ significantly increases the surface area and active sites of the composite, thereby providing more adsorption sites for pollutants and improving the photocatalytic performance [26].

#### 2.1.1. Optical Property Analysis of Materials

**Figure 6:**
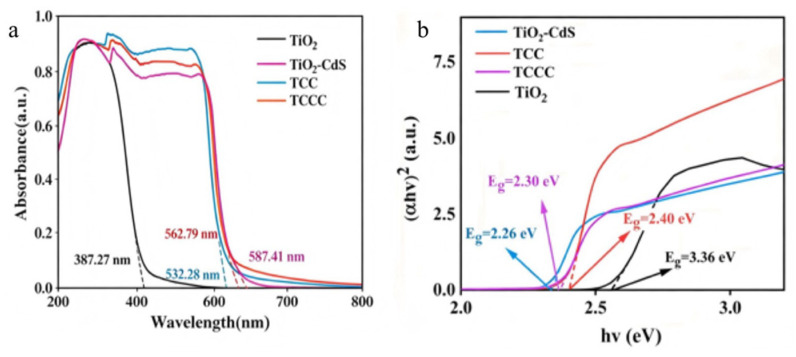
(**a**) UV-Vis absorption spectra and (**b**) corresponding Tauc plots of TiO_2_, TiO_2_-CdS, TCC, and TCCC.

The photocatalytic performance plays a crucial role in the degradation efficiency of MG. Thus, UV-Vis diffuse reflectance spectroscopy (DRS) was employed to obtain the optical energy data of different materials (Figure 6). The UV-Vis reflection data were measured using a Lambda UV-950 spectrophotometer (PerkinElmer, Waltham, MA, USA), where the absorbance α = 100−R (R is the reflectance). The optical band gaps of catalysts were determined by the Tauc plot method. According to the Tauc equation, the band gaps (Eg) of hybrid photocatalysts were calculated as Eg(TCCC) < Eg(TCC) < Eg(TiO_2_-CdS) < Eg(TiO_2_). Compared with TiO_2_, TCCC exhibits a smaller band gap, enhancing the light-harvesting ability of TiO_2_ and significantly inhibiting the recombination of photogenerated carriers, demonstrating the material’s superior photocatalytic performance.

**Figure 7:**
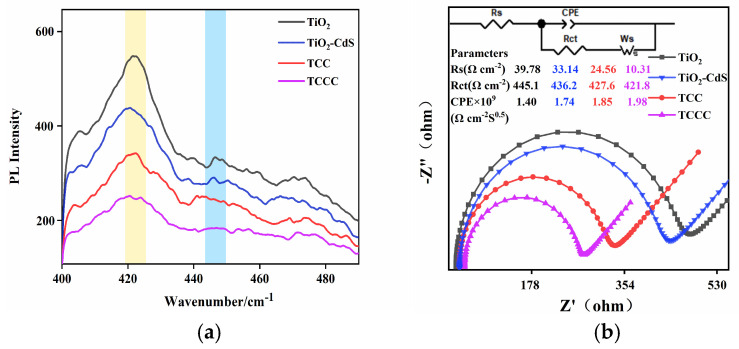
(**a**) PL spectra of TiO_2_, TiO_2_-CdS, TCC, and TCCC; (**b**) EIS spectra of TiO_2_, TiO_2_-CdS, TCC, and TCCC.

To further explore the contribution of charge transfer (CT) to the heterostructure, photoluminescence (PL) and electrochemical impedance spectroscopy (EIS) analyses were performed on the prepared substrates. Photoluminescence spectroscopy clearly demonstrates the persistence of charge recombination processes and carrier migration behavior. Steady-state photoluminescence spectra of the synthesized photocatalysts were recorded at an excitation wavelength of 325 nm. The 325 nm excitation wavelength was chosen because it matches the intrinsic light absorption range of the as-prepared photocatalysts, which can effectively excite photogenerated electron–hole pairs and acquire stable PL emission signals. As shown in Figure 7, all materials exhibit strong and broad PL signals with distinct peaks at approximately 427 nm and 473 nm. TiO_2_ shows the highest emission intensity among all samples, which may be directly correlated with its high electron–hole recombination rate. The PL emission band at 427 nm is attributed to interband recombination on the surface of anatase TiO_2_, while surface oxygen vacancies are identified at 473 nm. The intense peak at 427 nm indicates substantial generation and rapid recombination of electrons and holes. Coupling TiO_2_ with CdS and C_3_N_4_, along with the introduction of CDs, significantly reduces the emission intensity, suggesting that electrons in TiO_2_ can be accepted to delay electron–hole pair recombination—i.e., the separation efficiency of photoexcited electron–hole pairs is enhanced, inducing intermediate states in the TiO_2_ system [27]. Since PL spectral intensity correlates with e-h^+^ recombination, samples with lower PL intensity are expected to exhibit superior photodegradation performance. These results are consistent with the photodegradation of MG.

Electrochemical impedance spectroscopy (EIS) is typically used to analyze the transfer of photogenerated carriers in semiconductors, where a smaller arc radius in the high-frequency region indicates lower charge transfer resistance. EIS measurements were conducted using a standard three-electrode system under visible-light irradiation. A 300 W xenon lamp with a 420 nm cutoff filter served as the light source. The system consisted of a Pt electrode as the counter electrode, an Ag/AgCl electrode as the reference electrode, and a 0.5 mol/L Na_2_SO_4_ solution as the electrolyte, forming a standard three-electrode configuration for photocurrent and electrochemical impedance tests on different samples. As shown in Figure 7b, the arc radii in the EIS plots follow the order: TiO_2_ > TiO_2_-CdS > TCC > TCCC, indicating that TCCC exhibits the optimal photogenerated carrier transfer efficiency. The EIS results are consistent with the aforementioned PL findings. The EIS Nyquist plot was fitted with a standard equivalent circuit containing solution resistance and charge transfer resistance. A smaller semicircle radius represents lower interfacial resistance and more efficient separation of photogenerated electron–hole pairs.

#### 2.1.2. Evaluation of Material Catalytic Performance

The photocatalytic degradation performances of the as-prepared samples were evaluated using Malachite Green (MG) as the target pollutant under visible light irradiation. MG is a typical highly toxic and refractory triphenylmethane dye commonly found in aquaculture and industrial wastewater [28]. The detailed experimental procedures are provided in the Experiments and Methods section.

**Figure 8:**
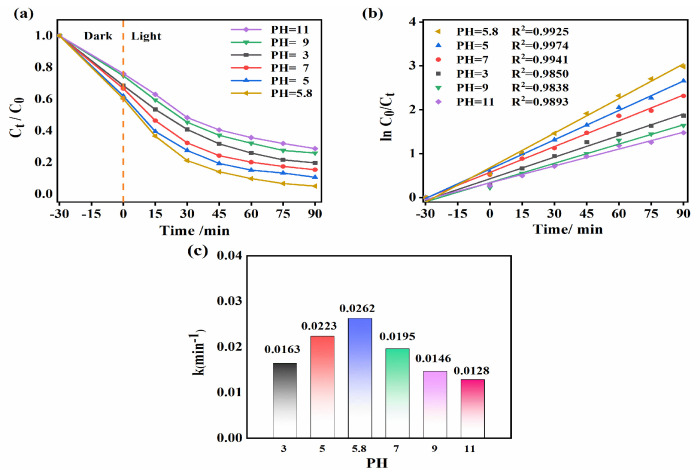
(**a**) Removal efficiency and (**b**) rate constant of MG by TCCC at different pH values. (**c**) Reaction rate constants (K) at different pH values.

We investigated the effect of different pH values on the degradation of MG by TCCC, as shown in Figure 8. The effect of strongly acidic conditions (pH = 3) on MG degradation is not significant, and the removal efficiency is similar to that under other acidic conditions. When pH = 11, the removal rate of MG decreases from 39.12% to 74.36% within 20 min, likely because the high concentration of OH- in the strongly alkaline environment inhibits the generation of ·OH, thus adversely affecting the degradation process. The change in pH from 5 to 7 does not cause significant variations in the MG removal rate or the k value (Figure 8b). In natural environments, most natural water bodies typically have a near-neutral pH between 6.5 and 8.5, where the system exhibits good MG removal capacity, indicating that the TCCC system has strong anti-interference ability and adaptability. We selected pH = 5.8, simulating the target pollutant solution, as the optimal pH condition for subsequent experiments.

**Figure 9:**
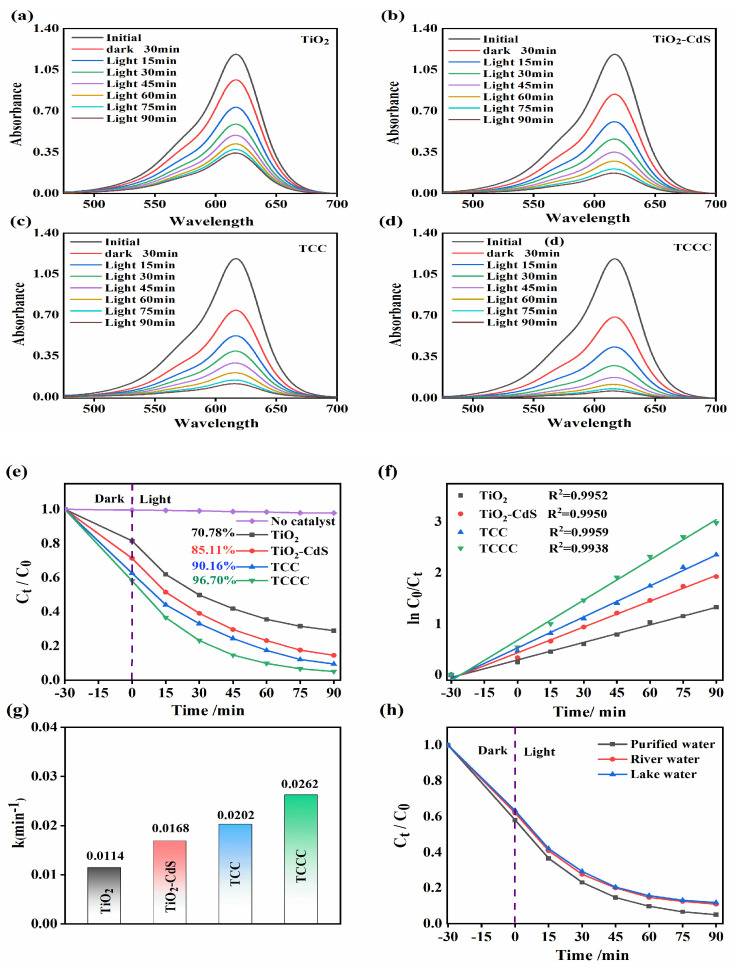
(**a**–**d**) UV-Vis spectra of MG degradation by TiO_2_, TiO_2_-CdS, TCC, and TCCC; (**e**) photocatalytic degradation efficiency; (**f**) corresponding kinetic rate constants; (**g**) comparison of kinetic rate constants; (**h**) degradation performance in different water matrices.

The photocatalytic performance of the prepared samples was evaluated by the decolorization efficiency of MG under visible light at room temperature and initial pH. As shown in Figure 9a–d, the order of photocatalytic degradation rates of MG decreases as: TCCC > TCC > TiO_2_-CdS > TiO_2_. Compared with other samples, TCCC exhibits the highest photocatalytic degradation efficiency for MG, reaching 96.70% (Figure 9e). Generally, a larger k value corresponds to a higher degradation rate and reaction rate. In Figure 9f, the calculated k values for different samples are 0.0114 min^−1^, 0.0168 min^−1^, 0.0202 min^−1^, and 0.0262 min^−1^, respectively. The results show that TCCC has the fastest degradation rate, indicating that the coupling of CdS, C_3_N_4_, and the introduction of CDs enables more efficient degradation of MG. The degradation results are consistent with the PL and EIS data, where the four-component TCCC significantly reduces the electron–hole recombination rate, and numerous active sites effectively initiate free radicals. We further analyzed the effect of photocatalytic material TCCC on the degradation of MG in different water sources to evaluate its practical application capability. The degradation efficiencies of the composite for MG in pure water, river water, and lake water were 96.70%, 94.36%, and 73.79%, respectively (Figure 9g,h). The composite catalyst still showed relatively high removal rates of MG in different water samples, confirming that TCCC has excellent degradation capacity and remediation potential for actual water samples.

Compared with previously reported photocatalysts for MG degradation, the TCCC composite prepared in this work shows higher degradation efficiency (96.70% within 90 min), larger reaction rate constant (0.0262 min^−1^), wider applicable pH range, and better stability after recycling. The results confirm that the multi-heterojunction structure and natural carbon quantum dots synergistically promote charge separation, enhance visible light absorption, and provide more active sites, thus endowing TCCC with superior photocatalytic performance.

#### 2.1.3. Investigation of Material Universality and Stability

**Figure 10:**
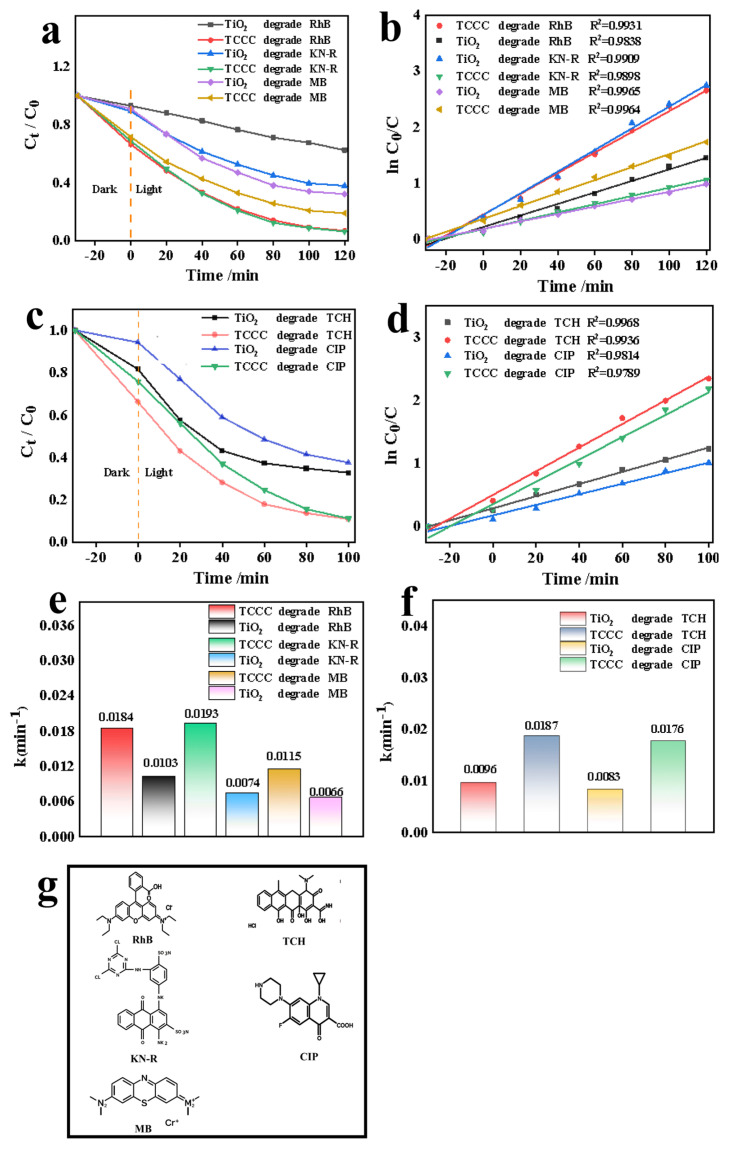
(**a**) Degradation curves of RhB, KN-R, and MB over TiO_2_ and TCCC; (**b**) corresponding kinetic rate constants; (**c**) degradation curves of TCH and CIP over TiO_2_ and TCCC; (**d**) corresponding kinetic rate constants; (**e**) comparison of rate constants for dye degradation; (**f**) comparison of rate constants for antibiotic degradation; (**g**) Chemical structures of the target pollutants.

Since real environments often contain dyes with diverse structures, catalytic systems capable of degrading only single-type organic dyes cannot meet practical needs. Therefore, in addition to MG, two dyes with different structures were selected to evaluate the TCCC system. As shown in Figure 10a, the TCCC system exhibits good removal efficiency for different dyes under optimal pH conditions. In Figure 10b, the calculated k values for various samples all conform to the first-order reaction kinetic equation, demonstrating the material’s good universality. Among them, KN-R was included as a representative anionic dye for comparative evaluation, further verifying the broad-spectrum degradation capability of TCCC.

The abuse and improper treatment of antibiotics lead to large amounts of drugs being directly discharged into natural environments such as water bodies and soil without complete absorption or degradation by organisms. These antibiotic residues are difficult to naturally eliminate, gradually accumulating and diffusing to form persistent pollution sources. Antibiotic residues in water may enter crops and drinking water sources through irrigation and drinking, further exacerbating environmental pollution. Thus, two typical antibiotics were selected to investigate the TCCC system: the quinolone antibiotic ciprofloxacin and the tetracycline antibiotic tetracycline hydrochloride. As shown in Figure 10c, the system demonstrates excellent degradation efficiency for both antibiotics under optimal conditions. The calculated k values in Figure 10d fit the first-order reaction kinetic equation, indicating the system’s capability to efficiently remove different types of dyes and antibiotics. These results confirm the versatility of the TCCC system and highlight its promising application prospects in actual water body degradation. Based on the literature, the photocatalytic degradation of these dyes and antibiotics mainly proceeds through ring-opening, deamination, dechlorination, and hydroxylation reactions, producing small-molecule intermediates, which can be further mineralized into CO_2_, H_2_O, and inorganic ions.

**Figure 11:**
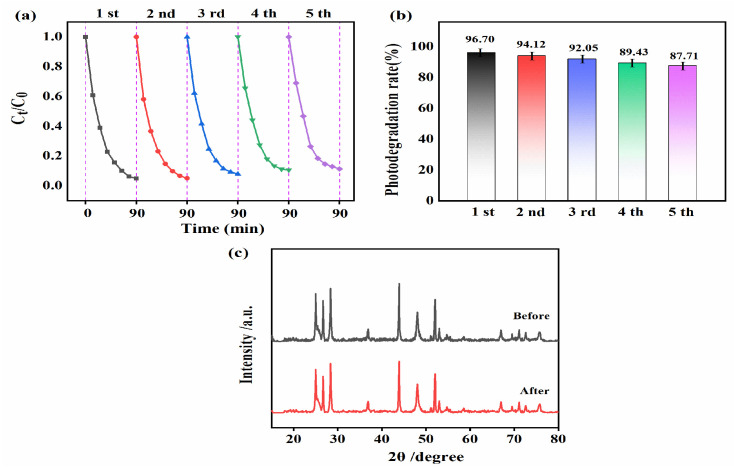
(**a**,**b**) Catalyst recycling tests; (**c**) XRD patterns of TCCC before and after 5 cycles.

The reusability of the catalyst is a critical factor in material stability studies and an important indicator for practical applications. Therefore, we investigated the recycling of the catalyst after MG removal. The MG removal performance slightly decreased with an increase in the number of cycles. After five cycles, the MG removal rate remained at 88.25% (Figure 11). The slight degradation in performance is attributed to intermediates generated during the degradation process possibly covering the catalyst surface, preventing some active sites from attaching to the substrate. Additionally, the catalyst recovery process also contributes to the reduced degradation performance. The above results demonstrate that the TCCC catalyst exhibits better comprehensive catalytic performance than its single-component and binary composite counterparts.

#### 2.1.4. Discussion on Photocatalytic Mechanism of the Composite Material

Investigating the effect of different catalysts on MG degradation aims to reveal the action mechanism of the TCCC catalytic system. To identify the main reactive species involved in the photocatalytic degradation of MG, isopropanol (IPA), ethylenediaminetetraacetic acid (EDTA), and benzoquinone (BQ) were selected as scavengers for ·OH, h^+^, and ·O_2_^−^, respectively.

**Figure 12:**
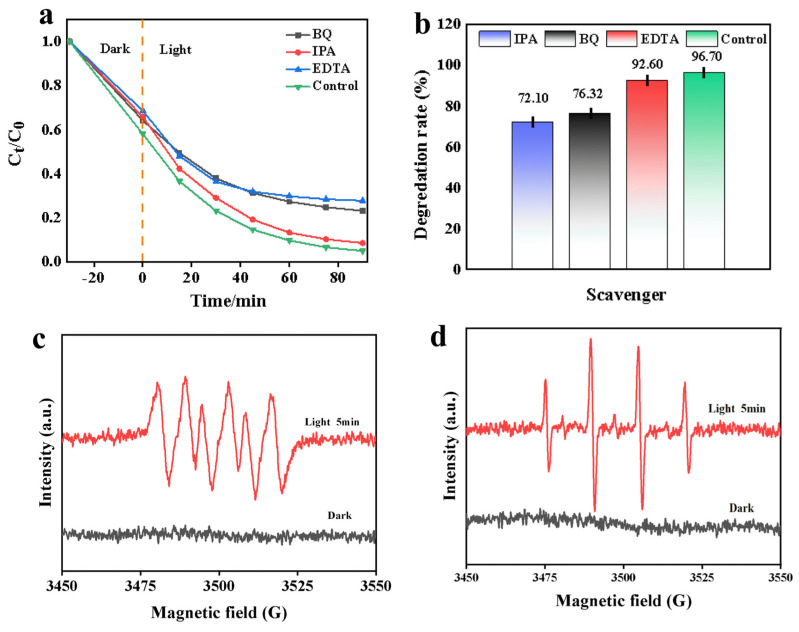
(**a**,**b**) Photocatalytic degradation efficiency of MB by different scavengers under visible light as a function of time; (**c**,**d**) EPR spectra of ·O_2_^−^ and ·OH generated in the system before and after light irradiation.

As shown in Figure 12a, the degradation efficiency of MG by TCCC reaches 96.70% without any scavengers. When isopropanol (IPA), benzoquinone (BQ), and ethylenediaminetetraacetic acid (EDTA) are added as scavengers for ·OH, ·O_2_^−^, and h^+^, respectively, the degradation efficiencies decrease to 72.10%, 76.32%, and 92.60%. The significant inhibition of photocatalytic degradation upon adding BQ to scavenge ·O_2_^−^ indicates that ·O_2_^−^ is the primary reactive species. Meanwhile, the addition of IPA also reduces the reaction rate, suggesting that ·OH plays an important role in the photocatalytic process. The degradation rate shows no significant decrease after adding EDTA, implying that the elimination of h^+^ has little effect on the photocatalytic reaction. The degradation efficiency with IPA is higher than that with EDTA and BQ, confirming that ·OH is the dominant reactive species in the photocatalytic degradation of MG. To further verify the presence of ·OH and ·O_2_^−^, electron paramagnetic resonance (EPR) spectroscopy was performed. As shown in Figure 12c,d, no signals of ·O_2_^−^ or ·OH were detected in the dark, while distinct signal peaks appeared under 5 min of light irradiation, confirming the generation of ·O_2_^−^ and ·OH [29].

**Figure 13:**
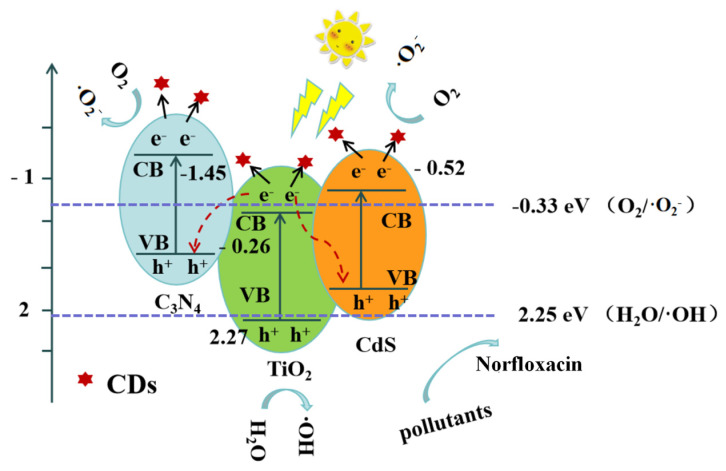
Photocatalytic mechanism of the composite material for MG degradation under visible light irradiation based on Z-scheme heterojunction.

The possible photocatalytic mechanism of the heterostructure for target pollutant degradation is illustrated in Figure 13. The conduction band (CB) and valence band (VB) potentials of TiO_2_, CdS and g-C_3_N_4_ were calculated based on their UV–Vis DRS band gaps and standard flat-band potential values. Under simulated solar light irradiation, electrons (e^−^) in the valence bands (VB) of TiO_2_, CdS, and g-C_3_N_4_ are excited to their conduction bands (CB), generating corresponding holes (h^+^) in the VB. When TiO_2_-CdS nanoparticles are uniformly deposited on g-C_3_N_4_, a double Z-scheme heterostructure is formed. This mechanism is supported by the UV–Vis DRS, PL and EIS results, confirming the Z-scheme charge transfer pathway. The contact interface between the two semiconductors drives e^−^ in the CB of TiO_2_ to migrate to the VB of CdS and g-C_3_N_4_, suppressing the recombination of photogenerated e^−^-h^+^ pairs and significantly enhancing photocatalytic activity for efficient pollutant degradation [30].

The e^−^ in the CB of TiO_2_ transfer to the VB of g-C_3_N_4_ and CdS. The conduction band potentials (ECB) of CdS and g-C_3_N_4_ are −1.45 eV and −0.52 eV, respectively, which are more negative than the redox potential of O_2_/·O_2_^−^ (−0.33 eV). Thus, e^−^ accumulated in the CB of CdS and g-C_3_N_4_ are captured by O_2_ to generate ·O_2_^−^ for pollutant catalysis [31,32]. Meanwhile, the ECB of TiO_2_ is 2.27 eV, higher than the redox potential of H_2_O/·OH (+2.25 eV), enabling h^+^ in the VB of TiO_2_ to react with OH^−^ to produce ·OH. Additionally, CQDs act as electron reservoirs, capturing photogenerated e^−^ to facilitate efficient separation of e^−^-h^+^ pairs and suppress their recombination, further enhancing the catalytic activity of the material. Although detailed intermediate identification is not the focus of this work, the results confirm that TCCC can effectively degrade and partially mineralize the target pollutants.

## 3. Experiments and Methods

### 3.1. Materials

All chemical reagents were of analytical grade and used without further purification. Tetrabutyl titanate (≥99.0%), cadmium nitrate tetrahydrate (Cd(NO_3_)_2_·4H_2_O, ≥99.0%), sodium sulfide nonahydrate (Na_2_S·9H_2_O, ≥98.0%), urea (≥99.0%), ethylene glycol (≥99.5%), nitric acid (70%), sodium hydroxide (≥96.0%), ethanol (≥99.7%), isopropanol (≥99.7%), ethylenediaminetetraacetic acid (≥99.0%), and benzoquinone (≥98.0%) were purchased from Sinopharm Chemical Reagent Co., Ltd. (Shanghai, China). Deionized water (18.2 MΩ·cm) was used throughout the experiments. Fresh orange peels were collected from locally purchased oranges (origin: Fujian Province, China).

In this study, the involved pollutants and their unified abbreviations are defined as follows: Malachite Green (MG), Methylene Blue (MB), Methylene Green (MGn), Tetracycline Hydrochloride (TCH), Ciprofloxacin (CIP), and Reactive Brilliant Blue KN-R (KN-R). Methylene Green is presented with its full name throughout the text to avoid abbreviation confusion.

### 3.2. Synthesis of Anatase TiO_2_

Anatase-phase TiO_2_ nanoparticles (TiO_2_ NPs) were synthesized via a classic solvothermal method. First, Solution A was added dropwise to Solution B at a rate of 2–3 drops per second with vigorous stirring. Solution A consisted of 5 mL tetrabutyl titanate and 5 mL ethanol, while Solution B was a mixture of 20 mL ethanol, 1 mL 70% nitric acid, and 5 mL deionized water. After the dropping process, stirring was continued for 1 h. The mixed solution was then transferred to a hydrothermal reactor and reacted at 160 °C for 6 h. Following heating, the reactor was allowed to cool to room temperature. The resulting white precipitate was collected by filtration, washed three times with deionized water and ethanol alternately, and then dried in an oven at 70 °C for 12 h. Subsequently, the dried product was calcined at 500 °C for 2 h to obtain pure anatase TiO_2_.

The hydrothermal synthesis of anatase TiO_2_ in this work was specifically performed to obtain highly crystalline, uniform anatase TiO_2_ nanoparticles with clean surfaces and abundant hydroxyl groups, which is critical for subsequent heterojunction construction with CdS. Commercial anatase TiO_2_ usually suffers from agglomeration, broad particle size distribution, and residual impurities, which would weaken the interfacial interaction between TiO_2_ and CdS and reduce charge separation efficiency. By using in-house synthesized TiO_2_, we ensured good dispersibility and strong interface coupling in TiO_2_-CdS, which is essential for forming efficient photocatalytic heterostructures.

### 3.3. Synthesis of CdS Monomer

0.8158 g of Cd(NO_3_)_2_·4H_2_O was dissolved in a mixture of 20 mL anhydrous ethanol and 5 mL deionized water. After thorough mixing, 0.6352 g of Na_2_S·9H_2_O was added, and the solution was sealed and stirred for 20 min. The resulting yellow precipitate was filtered, washed with deionized water and ethanol three times, and then transferred to a stainless-steel autoclave and subjected to a hydrothermal process at 160 °C for 6 h in a forced-air drying oven. After natural cooling, the product was poured into a watch glass and dried at 80 °C for 6 h. The obtained solid particles (CdS precursor) were calcined at 500 °C for 2 h to yield the CdS monomer.

### 3.4. Synthesis of TiO_2_-CdS NPs

The synthesis of TiO_2_-CdS nanoparticles (NPs) was modified based on the method reported by Abdelmoneim et al. [33]. A one-pot hydrothermal approach was employed: 4.5 mL of tetrabutyl titanate and 0.6352 g of Na_2_S·9H_2_O were mixed with 4.5 mL anhydrous ethanol, then added dropwise to a stirred solution containing 0.25 mL concentrated HNO_3_, 0.8158 g Cd(NO_3_)_2_·4H_2_O, 20 mL anhydrous ethanol, and 45 mL deionized water. After sealing and stirring for 2 h, the yellow precipitate was filtered and washed with deionized water three times, then transferred to an autoclave for hydrothermal treatment at 160 °C for 6 h. Following cooling and drying at 80 °C for 6 h, the TiO_2_-CdS precursor was calcined at 500 °C for 2 h to obtain TiO_2_-CdS NPs.

In this study, commercially available anatase TiO_2_ was not employed as a control sample for the synthesis of TiO_2_-CdS, because our primary aim was to construct a well-defined heterostructure based on uniform, impurity-free in-house synthesized TiO_2_. A systematic comparison between homemade and commercial TiO_2_ is beyond the scope of the present work and will be considered in future studies. The molar ratio of Ti to Cd in the TiO_2_-CdS composite was 1:1.

The photocatalytic degradation experiments were performed under a simulated sunlight irradiation system. The light source covered a wavelength range of 320–780 nm with stable light intensity throughout the reaction. The catalyst dosage and pollutant solution volume were kept constant for all tests to guarantee experimental reproducibility and comparability.

### 3.5. Preparation of TCC Composite

The as-synthesized TiO_2_-CdS NPs (0.2 g) were dispersed in 0.1 mol/L NaOH solution [34], stirred for 6 h to remove surface impurities, and dried. The product was then ground with 0.4 g urea and calcined in a covered crucible at 500 °C for 2 h, yielding the TiO_2_-CdS-C_3_N_4_ (TCC) composite. The molar ratio of TiO_2_:CdS:g-C_3_N_4_ in the TCC composite was 1:1:2.

### 3.6. Preparation of TCCC Composite

Orange peel was thoroughly washed, air-dried at room temperature for 48 h [35], and then ground into fine powder using an agate mortar. A 1 g portion of the powder was mixed with 10 mL ethylene glycol and microwaved (900 W, Midea, Foshan, China) for 1 min. The resulting dark-brown solution, indicating CQDs formation, was centrifuged at 15,000 rpm. The supernatant was purified via dialysis (molecular weight cutoff = 1000) for 12 h to remove insoluble residues and small molecules, then dried. A calculated amount of CQDs was mixed with TCC (nCQDs:nTi = 5%) in 20 mL deionized water and stirred at room temperature for 24 h. The mixture was washed alternately with deionized water and ethanol three times, then dried at 70 °C for 6 h to obtain the TCCC composite containing natural carbon quantum dots.

The nominal molar ratio of TiO_2_ to CdS to g-C_3_N_4_ in the designed TCCC composite was 1:1:2, and the molar ratio of CQDs to TiO_2_ was controlled at 5%.

### 3.7. Characterization

Powder X-ray diffraction (XRD) patterns were recorded on a Bruker D8 Advance diffractometer (Bruker, Karlsruhe, Germany; Cu Kα radiation, λ = 1.5406 Å, 40 kV, 40 mA) in the 2θ range of 10–80° with a scan rate of 5°/min. Fourier transform infrared (FTIR) spectra were obtained using a Nicolet iS50 spectrometer (Thermo Fisher Scientific, Waltham, MA, USA; KBr pellet method) in the range of 400–4000 cm^−1^. Scanning electron microscopy (SEM) images were captured on a Hitachi SU8010 field-emission SEM (Hitachi, Tokyo, Japan), and energy-dispersive X-ray spectroscopy (EDS) was performed using an Oxford X-max detector (Oxford Instruments, Abingdon, UK). X-ray photoelectron spectroscopy (XPS) was conducted on a Thermo Scientific K-Alpha^+^ spectrometer (Thermo Fisher Scientific, Waltham, MA, USA; Al Kα radiation, hν = 1486.6 eV), and binding energies were calibrated with C 1s at 284.8 eV. N_2_ adsorption–desorption isotherms (BET) were measured on a Micromeritics ASAP 2460 analyzer (Micromeritics, Norcross, GA, USA) at 77 K; samples were degassed at 150 °C for 6 h prior to measurement. UV-Vis diffuse reflectance spectra (UV-Vis DRS) were recorded on a PerkinElmer Lambda 950 spectrophotometer (PerkinElmer, Waltham, MA, USA) equipped with an integrating sphere, using BaSO_4_ as a reference. Photoluminescence (PL) spectra were collected on a Hitachi F-7000 fluorescence spectrophotometer (Hitachi, Tokyo, Japan) at an excitation wavelength of 325 nm. Electrochemical impedance spectroscopy (EIS) was performed on a CHI660E electrochemical workstation (Chenhua, Shanghai, China) using a standard three-electrode system with a 300 W Xe lamp (λ ≥ 420 nm) as the light source; the working electrode was prepared by coating sample slurry onto FTO glass, with Ag/AgCl as the reference electrode and Pt wire as the counter electrode in 0.5 mol/L Na_2_SO_4_ electrolyte. Electron paramagnetic resonance (EPR) spectra were recorded on a Bruker EMXplus spectrometer (Bruker, Karlsruhe, Germany) at room temperature using DMPO as a spin-trapping agent.

Photocatalytic degradation experiments were carried out in a 100 mL quartz beaker as the photoreactor. A 300 W xenon lamp (PerfectLight, PLS-SXE300, Beijing, China) equipped with a 420 nm cutoff filter was used as the visible light source (λ ≥ 420 nm). In each experiment, 20 mg of photocatalyst was dispersed in 100 mL of 10 mg/L MG aqueous solution. Prior to illumination, the suspension was stirred in the dark for 30 min to establish adsorption–desorption equilibrium. During irradiation, 3 mL aliquots were taken at regular intervals (every 30 min), and the catalyst particles were removed by centrifugation (10,000 rpm, 5 min). The concentration of MG was analyzed using a UV-Vis spectrophotometer (Shimadzu UV-2600, Shimadzu, Kyoto, Japan) by monitoring the absorbance at 617 nm.

## 4. Conclusions

In this work, TiO_2_-CdS nanoparticles (NPs) were synthesized via a one-pot hydrothermal method, loaded onto g-C_3_N_4_ with a large specific surface area, and integrated with natural green carbon quantum dots (CQDs) derived from orange peel as electron reservoirs, successfully fabricating the TCCC composite photocatalyst. The prepared TCCC composite forms a double Z-scheme heterojunction, which effectively promotes the separation and migration of photogenerated carriers, reduces the recombination rate of electrons and holes, and extends the absorption range of visible light. The synergistic effect among TiO_2_, CdS, g-C_3_N_4_ and CQDs is the core reason for the significant improvement of photocatalytic activity. The properties and photocatalytic applications of the prepared material were analyzed, and the results are as follows: Characterization techniques including X-ray diffraction (XRD), Fourier transform infrared spectroscopy (FTIR), Brunauer–Emmett–Teller (BET) analysis, scanning electron microscopy (SEM), and X-ray photoelectron spectroscopy (XPS) confirmed the successful synthesis of the TCCC photocatalyst. Optical properties were investigated by UV-Vis diffuse reflectance spectroscopy, photoluminescence (PL) spectroscopy, and electrochemical impedance spectroscopy (EIS). The UV-Vis results showed that TCCC enhances visible-light utilization, addressing the wide bandgap limitation of single-component TiO_2_. PL and EIS analyses indicated that constructing TCCC improves charge transfer efficiency, alters the migration pathway of photogenerated carriers, and enhances pollutant degradation efficiency. In photocatalytic activity tests, the degradation of organic dye malachite green (MG) by TCCC was evaluated, along with its universality and stability. The results demonstrated TCCC’s practical application potential for treating organic wastewater, exhibiting excellent degradation performance toward other dyes (rhodamine B, KN-R, methylene blue) and two antibiotics (ciprofloxacin, tetracycline hydrochloride). The excellent photocatalytic performance benefits from the rational combination of different functional components: CdS narrows the band gap and enhances visible light response; g-C_3_N_4_ provides a large specific surface area and abundant active sites; CQDs act as an electron reservoir to capture photogenerated electrons and further suppress charge recombination. These advantages make the TCCC composite possess higher photocatalytic efficiency than single and binary materials. After five cycles, TCCC maintained a high degradation rate for MG, confirming its recyclability and stability. Finally, radical trapping experiments, combined with all the characterization results, were used to deduce the degradation mechanism of target pollutants by TCCC.

## Data Availability

The data presented in this study are available upon request from the corresponding authors.

## References

[1] Heris S.Z., Khaniani P.B., Mousavi S.B. (2025). Photocatalytic degradation of co-amoxiclav using hybrid TiO_2_/ZnO nanoparticles: Experimental and optimization. J. Water Process Eng..

[2] Qi F., Yang Z., Qiu Q., Wang Y., Li H. (2022). Defective TiO_2_ with increased photocatalytic activity synthesized by the TiO_2_/Ti interfacial reaction method. Surf. Interfaces.

[3] Zeshan M., Bhatti I.A., Mohsin M., Iqbal M., Amjed N., Nisar J., AlMasoud N., Alomar T.S. (2022). Remediation of pesticides using TiO_2_ based photocatalytic strategies: A review. Chemosphere.

[4] Jing Y.-N., Yin X.-L., Li L.-L., Wang Y.-L., Xue J., Xu Z.-F., Liu D.-Q., Chen C.-W., Liu X.-J., Liu E.-K. (2024). Fe-TiO_2-x_/TiO_2_ S-scheme homojunction for efficient photocatalytic CO_2_ reduction. J. Colloid Interface Sci..

[5] Jeon J.P., Kweon D.H., Jang B.J., Kim M.J. (2020). Enhancing the photocatalytic activity of TiO_2_ catalysts. Adv. Sustain. Syst..

[6] Xu C., Liu X., Zhang Y., Liao F., Hu S., Muneer A., Su R., Deng F. (2025). Fabrication of sulfur-vacancy-rich CdS for efficient photocatalytic degradation of ofloxacin. J. Environ. Chem. Eng..

[7] Jia Y., Zhang Y., Zhang X., Cheng J., Xie Y., Zhang Y., Yin X., Song F., Cui H. (2023). Novel CdS/PANI/MWCNTs photocatalysts for photocatalytic degradation of xanthate in wastewater. Sep. Purif. Technol..

[8] Fan Y., Yang R., Zhu R., Zhao H., Lu Q., Chen Z., Hu J. (2022). CdS-based artificial leaf for photocatalytic hydrogen evolution and simultaneous degradation of biological wastewater. Chemosphere.

[9] Zhang C., Li N., Chen D., Xu Q., Li H., He J., Lu J. (2021). The ultrasonic-induced-piezoelectric enhanced photocatalytic performance of ZnO/CdS nanofibers for degradation of bisphenol A. J. Alloys Compd..

[10] Fu W., Wang S., Zhang Y., Cheng B., Wu Y. (2025). 2D/2D F-doped TiO_2_/CdS S-scheme heterojunction photocatalyst for enhanced photocatalytic H_2_ generation. J. Mater. Sci. Technol..

[11] Jiang T., Wang Z., Wei G., Wu S., Huang L., Li D., Ruan X., Liu Y., Jiang C., Ren F. (2024). Defective high-crystallinity g-C_3_N_4_ heterostructures by double-end modulation for photocatalysis. ACS Energy Lett..

[12] Li J., Zhang M., Li X., Li Q.Y., Yang J.J. (2017). Effect of the calcination temperature on the visible light photocatalytic activity of direct contact Z-scheme g-C_3_N_4_-TiO_2_ heterojunction. Appl. Catal. B Environ..

[13] Cheng M., Li H., Wu Z., Yu Z., Tao X., Huang L. (2025). Synergistic effects of CQDs and oxygen vacancies on CeO_2_ photocatalyst for efficient photocatalytic nitrogen fixation. Sep. Purif. Technol..

[14] Syed N., Huang J., Feng Y. (2022). CQDs as emerging trends for future prospect in enhancement of photocatalytic activity. Carbon Lett..

[15] Yang W., Bu Q. (2024). Microsphere structure enhances the photocatalytic performance of TiO_2_-CdS heterojunction. Mater. Lett..

[16] He F., Zhu B., Cheng B., Yu J., Ho W., Macyk W. (2020). 2D/2D/0D TiO_2_/C_3_N_4_/Ti_3_C_2_ MXene composite S-scheme photocatalyst with enhanced CO_2_ reduction activity. Appl. Catal. B Environ..

[17] Orak C., Oğuz T., Horoz S. (2024). Facile synthesis of Mn-doped CdS nanoparticles on carbon quantum dots: Towards efficient photocatalysis. J. Aust. Ceram. Soc..

[18] Singh A., Ahmed A., Sharma A., Sharma C., Paul S., Khosla A., Gupta V., Arya S. (2021). Promising photocatalytic degradation of methyl orange dye via sol-gel synthesized Ag–CdS@ Pr-TiO_2_ core/shell nanoparticles. Phys. B Condens. Matter.

[19] Jiang X., Jiang H., Tang Y.M., Zhang H.Z., Yang L.B., Wang X.W., Zhao B. (2024). g-C_3_N_4_/TiO_2-X_ heterojunction with high-efficiency carrier separation and multiple charge transfer paths for ultrasensitive SERS sensing. Chin. Chem. Lett..

[20] Manchwari S., Khatter J., Chauhan R.P. (2022). Enhanced photocatalytic efficiency of TiO_2_/CdS nanocomposites by manipulating CdS suspension on TiO_2_ nanoparticles. Inorg. Chem. Commun..

[21] Zhang M., Liu M., Jiang Y., Li J., Chen Q. (2020). Synthesis of immobilized CdS/TiO_2_ nanofiber heterostructure photocatalyst for efficient degradation of toluene. Water Air Soil. Pollut..

[22] Wang K., Liang L., Zheng Y., Li H., Niu X., Zhang D., Fan H. (2021). Visible light-driven photocatalytic degradation of organic pollutants via carbon quantum dots/TiO_2_. New J. Chem..

[23] Chen X., Peng X., Jiang L., Yuan X., Fei J., Zhang W. (2022). Photocatalytic removal of antibiotics by MOF-derived Ti^3+^-and oxygen vacancy-doped anatase/rutile TiO_2_ distributed in a carbon matrix. Chem. Eng. J..

[24] Deng X., Zhang D., Lu S., Bao T., Yu Z., Deng C. (2021). Green synthesis of Ag/g-C_3_N_4_ composite materials as a catalyst for DBD plasma in degradation of ethyl acetate. Mater. Sci. Eng. B.

[25] Farjadfar S., Ghiaci M., Kulinich S.A., Wunderlich W. (2022). Efficient photocatalyst for the degradation of cationic and anionic dyes prepared via modification of carbonized mesoporous TiO_2_ by encapsulation of carbon dots. Mater. Res. Bull..

[26] Saleh S.M., Albadri A.E.A.E., Ben Aissa M.A., Modwi A. (2022). Fabrication of mesoporous V_2_O_5_@ g-C_3_N_4_ nanocomposite as photocatalyst for dye degradation. Crystals.

[27] Xu L., Bai X., Guo L., Yang S., Jin P., Yang L. (2019). Facial fabrication of carbon quantum dots (CDs)-modified N-TiO_2-x_ nanocomposite for the efficient photoreduction of Cr (VI) under visible light. Chem. Eng. J..

[28] Viswanathan G., Solaiappan A., Thirumalairaj B., Krishnamoorthy U., Lakshmaiya N., Siddiqui M.I.H., Shah M.A. (2024). Improved photocatalytic properties of WO_3_ nanoparticles for Malachite green dye degradation under visible light irradiation: An effect of La doping. Open Chem..

[29] Chen J.J., Li Y.Y., Wang Y.P., Cui T.L., Jin A.L., Shang X.L., Qiao Y. (2021). Preparation and Photocatalytic Activity of C/g-C_3_N_4_/MoS_2_ Composites. Chin. J. Inorg. Chem..

[30] Li M., Lu Q., Liu M., Yin P., Wu C., Li H., Zhang Y., Yao S. (2020). Photoinduced charge separation via the double-electron transfer mechanism in nitrogen vacancies g-C_3_N_4_/BiOBr for the photoelectrochemical nitrogen reduction. ACS Appl. Mater. Interfaces.

[31] Liu Y., Tian J., Wei L., Wang Q., Wang C., Xing Z., Li X., Yang W., Yang C. (2021). Modified g-C_3_N_4_/TiO_2_/CdS ternary heterojunction nanocomposite as highly visible light active photocatalyst originated from CdS as the electron source of TiO_2_ to accelerate Z-type heterojunction. Sep. Purif. Technol..

[32] Gotipamul P.P., Vattikondala G., Rajan K.D., Khanna S., Rathinam M., Chidambaram S. (2022). Impact of piezoelectric effect on the heterogeneous visible photocatalysis of g-C_3_N_4_/Ag/ZnO tricomponent. Chemosphere.

[33] Abdelmoneim H.E.M., Wassel M.A., Elfeky A.S., Bendary S.H., Awad M.A., Salem S.S., Mahmoud S.A. (2021). Multiple applications of CdS/TiO_2_ nanocomposites synthesized via microwave-assisted sol–gel. J. Clust. Sci..

[34] Yang F., Zhu L., Xu Z., Han Y., Lin X., Shi J., Sun Z., Duan X. (2024). Multi-active photocatalysts of biochar-doped g-C_3_N_4_ incorporated with polyoxometalates for the high-efficient degradation of sulfamethoxazole. Environ. Pollut..

[35] Hu X., Li Y., Xu Y., Gan Z., Zou X., Shi J., Huang X., Li Z., Li Y. (2021). Green one-step synthesis of carbon quantum dots from orange peel for fluorescent detection of Escherichia coli in milk. Food Chem..

